# Multidrug‐Resistant *Salmonella* Spp. Isolated From Cloacal Swab of Healthy Layer Chickens and Egg Surface at Selected Farms in Bangladesh

**DOI:** 10.1155/vmi/5515539

**Published:** 2026-08-27

**Authors:** Most. Rafiya Akter, Md. Arefin Kallol, Shanzida Binte Alam, Md. Mahmudul Alam, Mohammad Ferdousur Rahman Khan, K. H. M. Nazmul Hussain Nazir, Md. Masudur Rahman, Marzia Rahman

**Affiliations:** ^1^ Department of Microbiology and Hygiene, Bangladesh Agricultural University, Mymensingh 2202, Bangladesh, bau.edu.bd; ^2^ Department of Surgery and Obstetrics, Bangladesh Agricultural University, Mymensingh 2202, Bangladesh, bau.edu.bd; ^3^ Department of Pathology, Sylhet Agricultural University, Sylhet 3100, Bangladesh, sau.ac.bd

**Keywords:** eggs, layer chickens, multidrug resistance, *Salmonella* spp

## Abstract

Multidrug‐resistant (MDR) *Salmonella* spp. is regarded as a perilous menace of public health significance. This research finding was carried out to investigate the prevalence of MDR *Salmonella* spp. in cloacal swabs of healthy layer birds and egg surface washing along with their resistance patterns and some associated resistance gene detection. In total, 120 samples consisting of 60 cloacal swabs and 60 eggs were collected from three different layer farms located at Mymensingh, Bangladesh, and subjected to various classical and molecular approaches. Antibiotic resistance patterns were investigated phenotypically using the Kirby–Bauer disk diffusion method and genotypically by detection of respective resistance genes. The overall prevalence rate of *Salmonella* spp. was 29% (95% CI: 18.077%−33.255%) and sample‐wise prevalence was 23% (95% CI: 14.415%−26.918%) in cloacal swab and 35% (95% CI: 22.043%−39.956%) in egg surface washing, respectively. Antibiotic susceptibility test revealed that the highest resistance was found to ampicillin 94.29% (95% CI: 86.65%–99.96%) followed by oxytetracycline 80.00%, (95% CI: 65.23%–98.03%), and erythromycin 65.72% (95% CI: 56.73%–75.41%), 45.71%, (95% CI: 33.039%–58.08%) to streptomycin, whereas the highest sensitivity was observed against florfenicol 74.29% (95% CI: 62.91%–85.28%), followed by ciprofloxacin (68.57%, 95% CI: 62.80%−73.66%), and sulphamethoxazole (68.57%, 95% CI: 53.48%–78.97%). The isolates were also found to be MDR as 80% (28/35) isolates (95% CI: 75.80%–86.69%) possessed a MAR index ranging between 0.33 and 0.89. On average, 19 isolates (54.28%; 95% CI: 26.90%−85.17%) were resistant to 5 – 8 antibiotics of seven classes, which is really alarming for public health significance. Based on PCR results, different resistant genes *blaTEM, tetA, aadA1, cat1,* and *flo-*R were amplified from 100%, 85.71%, 77.14%, 62.86%, and 37.14% of the isolates. Due to increased drug resistance, antibiotics and other antimicrobial drugs are becoming ineffective for the medication of both human and animal infections. Further investigations are also suggested to cope with antimicrobial resistance through this research study.

## 1. Introduction

As a zoonotic bacterial pathogen, *Salmonella* spp. bears great economic significance both in human health and livestock sectors. Multidrug‐resistant (MDR) *Salmonella* spp. has grown to become a global health problem because of its zoonotic importance [1, 2]. Moreover, avian salmonellosis is a fearful disease of poultry as it causes decreased production and remarkable financial losses in poultry farming worldwide [3].

Poultry eggs are an important animal protein source and nutritious foodstuffs for human that also fulfill the higher demand of egg industry. Improper handling of contaminated eggs and egg products may lead to salmonellosis and other food‐borne illness in human. Additionally, as a pathogenic bacterial agent, *Salmonella* spp. commonly leads to food‐borne infections and diseases [4, 5].

Usually, the eggs of laying hens become contaminated with *Salmonella* spp. horizontally from the feces of *Salmonella* spp.–affected hens or contaminated with environmental dust after oviposition and vertically before oviposition through the eggshell membrane, albumin, or egg yolk. On the other hand, the egg shells may be contaminated through two possible contamination routes such as vertical and horizontal [6, 7]. In layer farms, there is a high probability of contamination of eggs with *Salmonella* spp. through contaminated environmental materials such as water, poultry feed, feces, and other environmental materials. Nowadays, antimicrobial resistance (AMR) is a terrible detrimental forecast for global public health which causes the rise of untreatable infectious diseases and deaths [8]. *Salmonella* spp. is one of the most significant food‐borne pathogens to transmit AMR genes and may cause zoonosis [9]. It causes severe food poisoning, typhoid fever, gastroenteritis, and ultimately death worldwide [10].

Antibiotic usage, that is, inappropriate use in terms of abuse and misuse in poultry, is regarded as the significant cause which is stimulating the development and spread of antibiotic‐resistant microorganisms in the environment, agriculture, veterinary, and human. A large amount of antibiotics is used in farm animals and birds, especially in poultry, either as prophylaxis treatment or for growth promotion purposes, but in most cases of such use, is unnecessary that trigger the emergence of MDR *Salmonella* spp. [11, 12].

Various types of antimicrobial agents such as *β*‐lactamases, aminoglycosides, tetracyclines, macrolides, and quinolones are applied in poultry production either as growth promoters or as a therapeutic purpose that lead to the emergence of MDR bacteria [9]. The AMR creates a risky condition by decreasing the production line in poultry industry. Ingestion of poultry meat and eggs contaminated by MDR *Salmonella* spp. might cause serious human health problems, illness, and deaths [10].

Notably, in tracking and marking the spread of resistant bacterial strains, pinpointing the risk‐associated arears and addressing the controlling strategies, it is critically important to unveil the resistance profile of bacterial isolates and to investigate the broader epidemiological study [13–15]. Therefore, epidemiological investigation along with resistant profiling of *Salmonella* spp. may represent an actual scenario to define strategic controlling approach and an alarm of the associated risk factors concerning to public health significance. Therefore, this study has focused on resistance profiling of *Salmonella* spp. isolates from healthy layer chickens and egg surface in Bangladesh along with the epidemiological investigation. Additionally, for the controlling of the prevalence and circulation of MDR *Salmonella* spp. in the poultry sector, the regular investigation is mandatory. It is also important to determine the multiple antibiotic resistance (MAR) index of the prevalent *Salmonella* spp. as MAR indexing may reveal the potential risk level (high‐risk MAR index more than 0.2 and low‐risk MAR index less than 0.2) of the resistant bacteria [16].

Moreover, the investigation of the relationship between phenotypic antibiotic resistance patterns and genotypic detection of responsible antibiotic‐resistant genes of the prevailing MDR *Salmonella* spp. in healthy layer chickens and egg surface in Bangladesh is still not studied simultaneously, which is an important research question concerning to public health.

From this point of view, we investigated the prevalence of MDR *Salmonella* spp. phenotypically by the disc diffusion method, potential risk level of resistance and contamination by MAR indexing and genotypically by amplification of respective resistance genes circulating in layer chicken and egg washing of different areas of Mymensingh, Bangladesh.

## 2. Materials and Methods

### 2.1. Study Design

**FIGURE 1:**
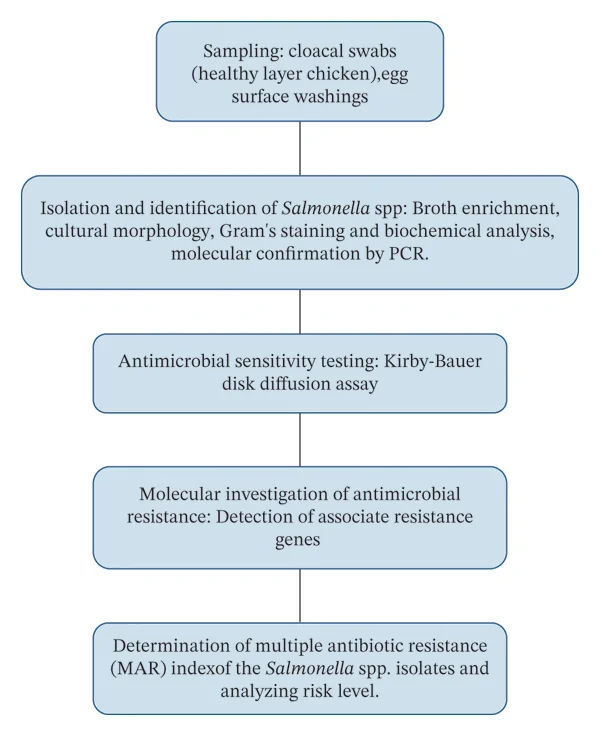
Brief schematic diagram of experimental pipeline.

This study was designed as a cross‐sectional survey which was combined of the strategy of cultural, biochemical, molecular (specific gene detection) identification, and antibiogram profiling of the target bacterial species (*Salmonella* spp.) from specific samples (cloacal swab of healthy layer chickens and egg surface) as a part of investigation of AMR considering public heath significance. A brief schematic diagram of the study is presented below in Figure 1.

### 2.2. Ethical Statement

The experimental design and the protocols applied in this research findings were certified by the ethics committee of the Department of Microbiology and Hygiene, Bangladesh Agricultural University (BAU), Approval Number: AWEEC/BAU/2018(20).

### 2.3. Study Area and Sampling

Three individual poultry farms from Mymensingh Sadar Upazila, Mymensingh, Bangladesh (24.75 N, 90.41 E), were selected for this study. The determination of sample size was conducted by the following equation based on the cross‐sectional survey [17] study design, *n* = Z^2^pq/d^2^, which was previously described by Thrusfeld [18], indicating *n* = probable sample size, *Z* = the standard normal deviation, (1.96 at 95% confidence level), *p* = prevalence (34.23% or 0.3423), *q* = (1‐p) = 1–0.3423 = 0.6577, and *d* = precision at 10% (*d* = 0.1). So *n* = (1.96)^2^ × 0.3423 × 0.6577/(0.1)^2^ = 86.49 ≈ 90. In Bangladesh, the average prevalence of *Salmonella* spp. was detected as 34.23% (95% CI: 23.73–46.56) after analyzing a recent 10‐year meta‐analysis of human, animal, and environment samples [19], as there is no individual published report on the prevalence of *Salmonella* spp. in cloacal swabs of healthy layer chickens along with egg surface washings in Bangladesh, and as this percentage covers both animal and environmental sample prevalence rate, this report was denoted as a reference for sample size calculation for this study. Furthermore, 30 (33%) more samples were taken as a part of additional sampling. As a result, in total 120 samples were collected (60 cloacal swabs and 60 egg surface washings) from BAU, Azad, and Abu Taleb Poultry Farms (cloacal swabs = 20 per farm; egg surface washings = 20 per farm). Cloacal swabs were taken aseptically from individual laying hen (apparently healthy, aged between 22 and 30 weeks) using sterile cotton buds. For egg sample washing samples, the eggs were collected by sterile hands from farms and kept instantly in a sterile plastic bag individually containing 10 mL of sterile phosphate buffer saline (PBS), rinsed for 5 min by shaking to wash the surface of the eggs thoroughly, and then the rinsed 10‐mL PBS was cultured overnight into 20 mL of nutrient broth for isolation of the *Salmonella* spp. [20].

### 2.4. Isolation and Identification of Salmonella Spp.

The collected cloacal swab samples using sterile cotton buds were directly inoculated into 10 mL of nutrient broth, and the egg washing samples (sample processing procedures are mentioned in *Study Area and Sampling* section) were inoculated into 20 mL of nutrient broth. Primarily, the inoculated samples into the nutrient broth were incubated at 37°C for overnight for bacterial enrichment. The overnight enriched samples were streaked on xylose lysine deoxycholate agar (XLD; Merch, Germany) plates and incubated for 24 h at 37°C for culturing of the desired bacterial species.

Black centered colonies with typical morphological appearance were primarily suspected as Salmonella spp. and were further defined by Gram’s staining and biochemical analysis such as urease test, methyl red, Voges–Proskauer, indole, and sugar fermentation. This isolation and identification procedures of *Salmonella* spp. was conducted following the standard methodology previously described [20, 21] with little modifications of the sample collection process.

The molecular confirmation of the primarily suspected isolates was performed by polymerase chain reaction (PCR) targeting the *invA* gene (Table 1), as the *invA* gene is a specific gene marker of *Salmonella* spp. that codes for an invasive protein which helps to invade the host epithelial cells [26].

**TABLE 1:** Primer sequences of different genes used in this study.

Name of gene	Primer sequence (5′–3′)	Amplicon size (bp)	References
invA	F‐ CGG TGG TTT TAA GCG TAC TCT T	796	[22]
	R‐ CGA ATA TGC TCC ACA AGG TTA		

blaTEM	F‐ CAT TTC CGT GTC GCC CTT AT	793	[23]
	R‐ TCC ATA GTT GCC TGACTCCC		

tetA	F‐ GGT TCA CTC GAA CGA CGT CA	577	[24]
	R‐ CTG TCC GAC AAG TTG CAT GC		

aadA1	F‐ TAT CAG AGG TAG TTG GCG TCA T	484	[25]
	R‐ GTT CCA TAG CG TTA AGG TTT CAT T		

cat1	F‐ CCTATAACCAGACCGTTCAG	508	[1]
	R‐ TCACAGACGGCATGATGAAC		

flo‐R	F‐ AAC CCG CCC TCT GGA TCA AGT CAA	548	[24]
	R‐ CAA ATC ACG GGC CAC GCT GTA TC		

*Note:* Here, *invA* gene is to identify *Salmonella* spp. The presence of *blaTEM* gene is associated with ampicillin/amoxicillin resistance, *tetA* gene is associated with tetracyclines resistance, *aadA1* gene is associated with streptomycin resistance, *cat1* gene is associated with chloramphenicol resistance, and *flo-R* gene is associated with florphenicol antibiotic resistance bacterial isolates.

### 2.5. Antimicrobial Sensitivity Testing

The antimicrobial sensitivity was tested by Kirby–Bauer disk diffusion assay [27] using Mueller–Hinton agar media (Oxoid, England), where standard protocols and guidelines were followed [28]. For this purpose, nine antibiotics (Oxoid, England) from seven commercially available and frequently applied antibiotic classes in poultry farms and human clinical treatment in Bangladesh [29], along with florfenicol (Fl), an exclusively used antibiotics in veterinary medicine all over the world [30], namely, amoxicillin (AMX, 10 μg), ampicillin (AMP, 10 μg), oxytetracycline (OXY, 30 μg), streptomycin (ST, 30 μg), ciprofloxacin (CIP, 5 μg), chloramphenicol (CHL 30 μg), Fl (30 μg), sulphamethoxazole (SUL, 25 μg), and erythromycin (ERY, 15 μg) were used. On the best of our knowledge, this is the first report of application of the antibiotic Fl in veterinary medicine, especially in aviary medicine in Bangladesh in recent years (confirmed by District livestock officers, Upazila livestock officers, and veterinary practitioners of different regions of Bangladesh.) and that is why we revealed the antibiogram profiling of prevalent *Salmonella* spp. (in this study) against Fl also. Based on the determination of zones of inhibition according to CLSI principles [28], the isolates which exhibited resistance to at least 3 classes of antibiotics were regarded as MDR [21].

### 2.6. Molecular Investigation of AMR

The conventional boiling method [31] with some modifications was followed for the extraction of genomic DNA of the isolated *Salmonella* spp. Briefly, a single colony of individual pure bacterial culture was mixed with 200 μL of sterile distilled water in an Eppendorf tube and boiled for 10 min. Then, the tube was instantly placed on ice and kept for 10 min for cold shock. In the next step, centrifugation was performed at 10,000 rpm for 10 min, and the supernatant was collected to use as template DNA for PCR. To investigate the resistance pattern, five antibiotic resistance genes were targeted as the genes are responsible for the development of resistance against the respective antibiotics, namely, *blaTEM* (for AMP/AMX resistance), *tetA* (for tetracyclines resistance)*, aadA1* (for ST resistance), *cat1* (for CHL resistance), and *flo-R* (for Fl resistance).

Selection of the resistance genes is directly linked with the investigation of phenotypic resistance profiling of the bacterial isolates and the regional antibiotics usage considering public health significance.

Therefore, AMX, AMP, OXY, ST, CIP, CHL, SUL, and ERY are commonly used antibiotics in Bangladesh in case of poultry farming and clinical medication for human [29], along with Fl, an exclusively used antibiotic in veterinary medicine worldwide [30], and the selected genes are responsible for or associated with the resistance of bacterial strains against those antibiotics individually.

A 25‐μL of PCR reaction volume was prepared following the procedure described by Alam et al., 2020, in case of each molecular amplification [21] (Table 2). Amplification of selected genes were performed using the primers listed in Table 1 with referred thermal profile of the regarding references. The PCR products were electrophoresed in 1.5% agarose gel along with a 100 base pair DNA Ladder (Promega, USA), and then gel documentation was performed (Alpha Innotech, Biometra, Germany).

**TABLE 2:** Component of PCR reaction volume for amplification of specific genes.

Component	Volume
PCR master mix 2X (Promega, USA)	12.5 μL
Forward primer (100 pmol)	1 µL
Reverse primer (100 pmol)	1 µL
Nuclease‐free water	5.5 µL
Genomic DNA template	5 µL
Total reaction volume	25 µL

In every step of lab experiment, *Salmonella enterica* strain MBR‐MFRK‐23 (characterized by Popy et al.) [32] was used a positive control for experimental validation and crosschecking our findings, although the experimental result of positive control is not mentioned in this research study.

### 2.7. Determination of MAR Index

Assessment of the MAR index determined for every isolate was performed by following the formula, MAR = a/b, where a’ denotes the number of antibiotics to which the test isolate exhibited resistance and b’ denotes the total number of antibiotics to which the test isolate has been assessed for susceptibility [33].

## 3. Results

### 3.1. Prevalence of *Salmonella* Spp.

Out of 120 samples, 35 isolates were identified as *Salmonella* spp. by cultural method, biochemical experiments, and molecular detection by PCR. The overall prevalence of *Salmonella* spp. was 29% (95% CI: 18.077%−33.255%). Sample‐wise, prevalence of *Salmonella* spp. in cloacal swab and egg washing were found as 23% (95% CI: 14.415%−26.918%) and 35% (95% CI: 22.043%−39.956%), respectively. The prevalence of *Salmonella* spp. in different samples is presented in Table 3. Primarily identified *Salmonella* spp. based on conventional tests (cultural method and biochemical experiments) were confirmed by PCR targeting *inv-A* gene, as this gene is specific for members of the genus *Salmonella* conserved among *Salmonella* serotypes [26].

**TABLE 3:** Prevalence of *Salmonella* spp. in cloacal swabs of layer chickens and egg surface washings at different farms in Mymensingh.

Sample source	Sample type	Sample size	Prevalence (%)	95% CI
Healthy layer chicken	Cloacal swabs	60	14 (23^a^)	14.415−26.918
	Egg surface washings	60	21 (35^b^)	22.043−39.956
	Overall count	120	35 (29)	18.077−33.255

*Note:* Here, the values with different superscripts (a, b) within the same variable differ significantly (*p* < 0.05). There is a statistically significant difference between the *Salmonella* prevalence rate of cloacal swabs and egg surface washings samples (*p* value = 0.0158).

It is notable, a 10‐year meta‐analysis of comparative data on the prevalence and resistance profiling of *Salmonella* spp. from human, animal, and environment samples represented that Bangladesh had the highest rate of *Salmonella* spp. prevalence (34.23%, 95% CI: 23.73–46.56) in South Asia. Pakistan also had a relatively higher prevalence rate (26.15%, 95% CI: 15.04–41.47). Out of all the South Asian countries, Sri Lanka had the lowest prevalence (2.04%, 95% CI: 0.51–7.79), according to only one study. In India, the prevalence rate was likewise lower at 3.04%, whereas Bhutan and Nepal had prevalence rate as 12.78% and 10.36% [19].

### 3.2. Phenotypic and Genotypic Resistance Profile of Isolated *Salmonella* Spp. to the Selected Antibiotics

Antimicrobial sensitivity test (AST) was performed to all the *Salmonella* spp. isolates by disc diffusion assay which evolved that, 94.29% (95% CI: 86.65%–99.96%) of *Salmonella* spp. isolates exhibited resistance to AMP; subsequently 80.00%, (95% CI: 65.23%–98.03%) to OXY; 65.72% (95% CI: 56.73%–75.41%) to ERY; 45.71%, (95% CI: 33.039%–58.08%) to ST and CHL; 31.43%, (95% CI: 23.671%–40.21%) to AMX and SUL; 28.57% (95% CI: 23.27%–34.73%) to CIP; and 20% (95% CI: 15.42%–28.57%) to Fl.

None of the isolates were found 100% sensitive to any antibiotics. However, 54.29% of the isolates (95% CI: 44.76%–63.89%) showed intermediately resistant to AMX.

**FIGURE 2:**
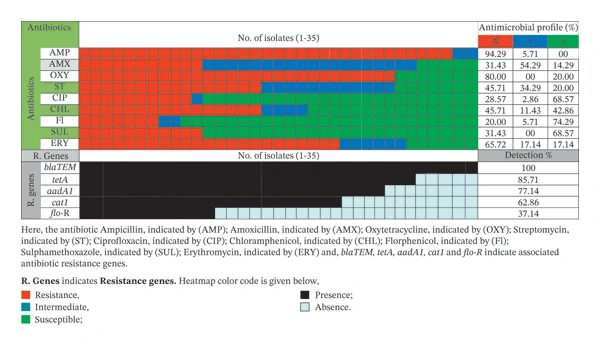
Heatmap with data table of antimicrobial resistance profiles of *Salmonella* spp. isolated from cloacal swab of healthy layers and egg washings.

Additionally, 74.29% (95% CI: 62.91%–85.28%) of the *Salmonella* spp. isolates revealed high sensitivity to Fl, CIP (68.57%, 95% CI: 62.80%–73.66%) and SUL (68.57%, 95% CI: 53.48%–78.97%) (Figure 2).

To validate the phenotypic resistance profile of the isolated *Salmonella* spp. simultaneously phenotypic and genotypic resistance status sketching were performed by AST and PCR using specific primer pairs of the resistance genes. All *Salmonella* spp. isolates were found to be harboring the beta‐lactamase–producing bla_TEM_ gene. Whereas 85.71% (95% CI: 79.87%–90.70%) harbor tetracycline resistance *tetA* gene, 77.14% (95% CI: 69.42%–83.84%) harbor ST resistance *aadA1* gene, 62.86% (95% CI: 54.46%–72.97%) bear CHL resistance *cat1* gene, and 37.14% (95% CI: 27.61%–49.87%) isolates found to be harboring Fl resistance *flo-*R gene (Figure 2).

### 3.3. Multidrug Resistance (MDR) Profile and MAR Index of *Salmonella* Spp. Isolated From Cloacal Swabs and Egg Surface of Layer Chickens

**FIGURE 3:**
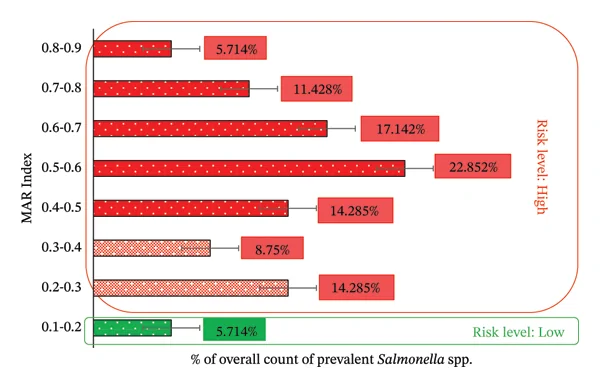
Risk factor levels of overall count of prevalent *Salmonella* spp. according to MAR index. Here, MAR index more than 0.2 was considered as at high‐risk level (for animal, human, or for both), and MAR index less than 0.2 was considered low‐risk level of the prevalent bacterial isolates. *X* axis represents MAR index (0.1–0.9), and *Y* axis represents % of overall count of prevalent *Salmonella* spp. (35 positive isolates from total cloacal swab and egg surface washing samples, which were considered as % of 100 in total calculative ratio).

In this study, a total of 9 antibiotics were used to analyze the phenotypic resistance pattern of the isolates. The phenotypic and genotypic resistance profiles along with MAR index are presented in Figures 2–3. The ranges of MAR indices of the isolates were 0.11–0.89. Phenotypically, out of 35 isolates, 28 *Salmonella* spp. isolates (80%; 95% CI: 75.80%–86.69%) were found to be MDR and were classified in the high‐risk level category (MAR index more than 0.2). Only two isolates (5.71%) showed resistance to one antibiotic and were classified in the low‐risk level category (MAR index less than 0.2). Although 5 isolates (14.29%) exhibited resistance to only two antibiotics, on average 19 isolates (54.28%; 95% CI: 26.90%−85.17%) were resistant to 5 – 8 antibiotics of seven classes, which is really alarming for public health significance (Figure 2–3).

Here, the antibiotic ampicillin, indicated by AMP; amoxicillin, indicated by AMX; oxytetracycline, indicated by OXY; streptomycin, indicated by (ST); ciprofloxacin, indicated by CIP; chloramphenicol, indicated by CHL; florphenicol, indicated by Fl; sulphamethoxazole, indicated by (SUL); erythromycin, indicated by (ERY) and, *blaTEM*, *tetA*, *aadA1*, *cat1*, and *flo-R* indicate associated antibiotic resistance genes.

## 4. Discussion

In the present study, investigation was conducted to ascertain the prevalence of MDR *Salmonella* spp. (from 60 cloacal swab samples of healthy layer chickens and 60 egg surface washing samples) circulating in the layer chicken farms in different regions of Mymensingh, Bangladesh. Because of the foremost concerns for public health, AMR situation, and the vibrant zoonotic and economic importance of *Salmonella* spp., regular tracing of the *Salmonella* prevalence and the trends of antibiotic resistance of the circulating isolates in poultry farming is essential for controlling risks.

Out of 120 samples (cloacal swabs and egg surface washings), 35 (29%, 95% CI: 18.08%–33.26%) were positive for *Salmonella* spp., with 80% (95% CI: 75.80%–86.69%) identified as MDR, where the *Salmonella* spp. prevalence in cloacal swabs and egg washings was 23% (95% CI: 14.42%–26.92%) and 35% (95% CI: 22.04%–39.96%), respectively. The overall prevalence of this study findings is higher than reports from Ethiopia (16.7%) in 2017 [34] and Egypt (19.3%) [35] in 2021, but similar to previous studies of Bangladesh (29%–31.25%) [36, 37] in 2020‐2021, and exceeds the pooled prevalence rate in India (18%) [38] from January 2010 to December 2023 in cloacal swab, feed, water, and attendant hand rinse samples collected from poultry farms. According to Mehmood et al., in 2024 [39], the prevalence rate of *Salmonella* spp. was 26.7% in unclean eggs in Pakistan, which is similar to our findings in Bangladesh. In Nepal, *Salmonella* prevalence in eggs was 2.5% [40] from a published data in 2025, much lower than that in Bangladesh. In Sri Lanka, *Salmonella* prevalence in eggshells and contents was 6.7% [41] in 2025, significantly lower than the findings in Bangladesh. *Salmonella* prevalence from cloacal swab samples of this study was lower than the reports from Bangladesh (32%) [42] in 2017 and Ecuador (33%) [43] in 2019 but higher than that from the United States (6%) [44] in a published report in 2018. In the egg washing samples, the prevalence (35%) was higher than previous reports from Bangladesh (28%) [42], China (27.3%) [45], and Thailand (15%) [46] from 2017–20122. So regional comparisons have revealed the higher prevalence of *Salmonella* spp. in poultry in Bangladesh than that in several neighboring countries, reflecting differences in poor hygiene practices, biosecurity standards, and antimicrobial usage.

Although, the stated variance in prevalence may be the consequence of differences in the sample type, region, breed, age of the birds, differences in flock management, hygienic measures, and biosafety measures [34]. In egg samples, the prevalence ratio was significantly higher than that in the cloacal swab samples. It might be due to the contamination of the egg surface with litter or feces during laying or after dropping to the floor in unhygienic conditions or from infected poultry [47], as García et al. [48] demonstrated that the cloaca is an emergent trace involved in the further infection of the egg.

Talukder et al. [19] reported that, over the past decade, Bangladesh had the highest prevalence of *Salmonella* spp. in poultry farms, meat, and eggs within South Asia. Our findings confirm that this high prevalence persists in recent years, posing significant public health and AMR concerns. Contributing factors likely include widespread and improper antimicrobial use, inadequate biosecurity and hygiene practices, and insufficient monitoring and enforcement of regulations. These conditions facilitate the persistence and spread of Salmonella, highlighting the urgent need for strengthened AMR control and farm‐level interventions. Investigation was performed on the isolates to determine susceptibility and resistance patterns by disc diffusion assay using 9 traditionally and most commonly used antibiotics belonging to 7 different classes. In this study, about 80% (95% CI: 75.80%–86.69%) of *Salmonella* isolates were detected as MDR which showed resistance to 3 to 8 antibiotics and according to the study of Abdi et al., the MDR percentage was 93.4% among the *Salmonella* spp. isolates from poultry in Ethiopia, which is almost in agreement with this study. The highest resistance was observed to AMP (94.29%), (95% CI: 86.65%–99.96%) which is almost similar to the findings by Abdi et al., 2017 in Ethiopia ((97.8%) [34], and the most susceptible antibiotic in this study was Fl which showed 74.29%, (95% CI: 62.91%–85.28%) sensitivity, followed by CIP and SUL which showed 68.57% sensitivity. Hosain et al. [49] in Bangladesh stated that *Salmonella* isolates from pigeons were resistant to AMX (90%), followed by AMP (80%) and sensitive to CIP (80%) followed by SUL (70%) and CHL (60%), which is aggregately similar to this study. The AMR patterns of *Salmonella* spp. isolates in this study are almost similar with most of the cases (some variations were also observed in few cases) of the broad‐spectrum data analysis on published reports on resistance profiling and patterns of *Salmonella* spp. in poultry (cloacal swabs, egg surface, feces, and hand wash) in Bangladesh [3] during the time span from 2011 to 2021.

It is remarkable that a certain number of *Salmonella* isolates (45.71%) showed resistance to CHL phenotypically, and 62.86% was detected genotypically by PCR. The phenotypic resistance of the isolates was confirmed by the amplification of 5 respective genes. It was also found that the detection rate of antibiotic resistance genes in the *Salmonella* spp. isolates was higher than the phenotypical detection rate by disc diffusion assay (Figure 2). It might be due to the acquiring of resistance genes that are not expressed phenotypically yet, but possibly in the future, they might be expressed phenotypically.

In this study, 94.29% of *Salmonella* isolates were identified as high‐risk level zoonotic bacterial pathogens (MAR index greater than 0.2), while only 5.71% of the isolates were classified as low‐risk level zoonotic bacterial pathogens (MAR index less than 0.2), which is a serious public health concern. However, MDR and MAR index findings of this study (Figure 2–3) are almost similar to the study of Abou Elez et al., in Egypt (MDR was observed in 75.7% *Salmonella* isolates from fecal swabs from laying hens, table eggs, and human stool samples with a MAR index ranging from 0.21 to 0.57) [35]. The possible causes of the higher percentage of MDR isolates with high MAR index may be linked to uncontrolled antibiotic usage, resulting in significant AMR associated risks for human, animal, or both.

So, in short, we focused on the cross‐sectional prevalence study of *Salmonella* spp. in layer birds and egg surface washings in selected regions of Bangladesh. But considering public health significance, the AMR profiling and MAR indexing have been also performed and aligned with the risk level, and the ARM profiling was performed both in phenotypic resistance profiling with responsible resistance gene detection for increasing the reliability of the findings.

Although we have conducted the study in a scientific way described previously in methodology, we concise some limitation of this study, such as along with the detection of *invA* gene to identify *Salmonella* spp. and *blaTEM*, *tetA*, *aadA1*, *cat1*, *flo-R* antibiotic resistance gene, more antibiotic resistance, and virulent genes were needed to be investigated. Partial or next generation sequencing (NGS) and data analysis would reveal more broad and significant validation of the study. Moreover, data collection of farm hygiene and waste management by individual interview of the farm owner would be additionally informative for this research investigation.

Beyond the limitations, the research findings will contribute to AMR surveillance and inform policy or veterinary practice by providing data on prevalence and MDR status of *Salmonella* spp. in layer farms (apparently healthy layer chickens as a MDR *Salmonella* spp. host) and eggs, unveiling the resistance level of prevalent *Salmonell*a spp. against available and commonly used or applied antibiotics in Bangladesh (both in veterinary and human practice), also contribute as an early‐warning for emerging high‐risk resistance genes to the AMR surveillance authority. Additionally, this study provides a message that, proper hygiene management should be applied strictly in all steps of egg transportation from layer farms to consumer’s tables to minimize or control the transmission of MDR *Salmonella* spp., as contaminated egg surfaces act as a carrier of MDR bacterial transmission. Moreover, random and misuses of antibiotics must be avoided in the field level also.

The antibiotic susceptibility should be demonstrated regularly to minimize the prevalence of MDR *Salmonella* spp. in chicken and eggs, as the hygiene practice in farm management and egg handling and transportation in Bangladesh are not followed properly. From egg collection after laying till consumption, the eggs are handled roughly in most of the cases. As a result, there is a considerable risk of *Salmonella* spp. transfer from farm to farm, markets, and even to consumers. Noticeably, as a high percentage of *Salmonella* spp. prevalence was detected in healthy layers and eggs, it might affect the poultry production in Bangladesh, and consumers may get infected by ingestion of contaminated eggs and meat. The authority should take action to constrict the unauthorized and abuse of antibiotics by farmers and clinicians and ensure hygiene practices in poultry farms, agriculture, and fisheries to minimize the *Salmonella* infection in human.

## 5. Conclusion

This research study findings revealed some warning facts of the prevalence of MDR *Salmonella* spp. with high MAR index circulating in some important location poultry farms in the Mymensingh region (near to BAU, which plays a central role in poultry research, disease diagnosis, vaccine development, and farmer training on antimicrobial uses) of Bangladesh. This incident indicates to the probable uncontrol and improper uses of antibiotics in poultry farms in Bangladesh and that is really concerning for potential public health risks specially in antibiotic resistance possibilities for both human and animal via zoonotic transmission. Widespread further investigations are also suggested, and AMR awareness should be drawn by regarding authorities strictly.

## Author Contributions

Conceptualization, research designing, validation, and visualization were performed by Md. Arefin Kallol, Mohammad Ferdousur Rahman Khan, K. H. M. Nazmul Hussain Nazir, Md. Mahmudul Alam, and Marzia Rahman; lab experiments, investigations, and preparing original draft were performed by Most. Rafiya Akter, Md. Arefin Kallol, and Shanzida Binte Alam; reviewing, editing, and data analyzing were performed by Md. Arefin Kallol, K. H. M. Nazmul Hussain Nazir, Md. Masudur Rahman, and Marzia Rahman; supervision was performed by Marzia Rahman. Resources and project administration were performed by Marzia Rahman.

## Funding

Funding was received from Bangladesh Agricultural University Research System (BAURES) for conducting this research work. Project Grant number: 2018/581/BAU.

## Disclosure

Final manuscript was approved by all authors for publication.

## Conflicts of Interest

The authors declare no conflicts of interest.

## Data Availability

The data used to support the research findings of this study are included within the article and are available to the corresponding author for consideration of request.
